# Use of Averaged Norbeck Social Support Questionnaire Scores

**DOI:** 10.5402/2011/567280

**Published:** 2011-06-07

**Authors:** Eileen Gigliotti, William Ellery Samuels

**Affiliations:** ^1^Department of Nursing, College of Staten Island and The Graduate Center, The City University of New York—CUNY 2800 Victory Boulevard, Staten Island, NY 10314, USA; ^2^The Graduate Center, The City University of New York—CUNY, 365 Fifth Avenue New York, NY 10016, USA; ^3^Department of Education, College of Staten Island, The City University of New York—CUNY, 2800 Victory Boulevard, Staten Island, NY 10314, USA

## Abstract

*Background*. Averaged Norbeck Social Support Questionnaire (NSSQ) support scores remove the influence of network size variability but may unduly lower scores for participants with large networks. *Objectives*. To evaluate the use of averaged NSSQ scores. *Method*. Pearson correlations determined if averaged scores decreased as network size increased across three samples. Then, Pearson correlations between a criterion variable and both averaged and raw support scores were computed along with the resultant power to detect a true effect. *Results*. Neither averaged total functional support nor averaged affect and affirmation scores decreased as sample size increased. However, averaged aid scores did decrease as network size increased. Power also increased markedly in all averaged versus raw scores except in averaged aid scores. *Discussion and Conclusions*. Use of averaged aid scores is not recommended. Use of all other averaged scores appears acceptable.

## 1. Introduction

The Norbeck social support questionnaire (NSSQ) [1, 2] is a network-based social support inventory. That is, unlike global support measures which assess overall perception of how supported one feels, the NSSQ asks participants to take detailed stock or inventory of (a) how many supportive network members they have and (b) how much affection, affirmation, and aid each network member provides. The strength of a network-based inventory is that it allows testing of multiple social support hypotheses, which are impossible to test using global measures of social support. Moving from general to specific, one can investigate the effects of total functional support (affect, affirmation, and aid) from the entire network, each of the three types of support from the entire network, total functional support from each network relationship (e.g., total functional support from spouse, friends, etc.), or each of the three types of support from each network relationship (e.g., affect from spouse). 

Despite the advantages of being a network based inventory, House and Kahn [3] argued that the NSSQ scoring system both creates extraneous variance and is a source of measurement error. They noted that network size varies considerably, because participants can nominate up to 24 network members. Thus, when participants' support ratings of network members are summed, NSSQ support scores may be confounded from extraneous variance from network size, and this is especially problematic when using total network scores versus specific network relationship scores. That is, because more network members implies more support, support scores from the entire network most heavily reflect *both* support ratings *and* number of supporters listed. Recognizing this problem, some investigators use averaged scores (support ratings divided by network size) to remove the influence of network size variability.

However, though effective in removing the effects of network size variability, as fully detailed below, Norbeck [4] discouraged this practice, cautioning that averaging can unduly lower scores of some participants with large networks. Though this may be true, to date, the effects of averaging on support scores as network size increases have not been investigated. Moreover, because extraneous variance due to network size variability in raw scores increases measurement error, averaged scores continue to be used. In fact, 23% of NSSQ-based studies published since Norbeck's caution in 1995 report averaged scores. 

The purpose of this paper is twofold: first, using three different data sets, we evaluate Norbeck's [4] concern by investigating whether averaged support scores do indeed decrease as network size increases. Second, we evaluate the statistical efficiency of averaged versus raw scores by comparing the powers of their correlations with a reasonable criterion variable. We then offer recommendations for the use of averaged scores based on these investigations.

## 2. Background

### 2.1. The NSSQ

The conceptual basis for the NSSQ [1, 2] is Kahn's [5] definition of social support, and it thus includes measures of functional support (affect, affirmation, and aid), network size, and network relationships. For this reason, the NSSQ was commended in House and Kahn's [3] classic work on social support concepts and measures, where they urged investigators to consider all three of these aspects of support, because network size is a “necessary condition and hence a partial determinant” (page 85) of network relationships and the types of support given. Due to its comprehensive scope and extensive ongoing psychometric evaluation [1, 2, 4, 6–8], it is one of the most widely used social support measures in nursing research. In fact, since its inception in 1981, the NSSQ has been used in over 250 studies published in peer-reviewed journals, and its use increases each year.

One reason for its widespread use is that unlike other network-based support inventories, the NSSQ is completed by the participant without input from an interviewer. This makes it ideal for use in large-scale studies such as mailed surveys. Because of this self-report feature, the NSSQ requires a unique layout. Specifically, participants are first asked to list from 1 to 24 network members “who provide personal support for you or who are important to you” and then specify their relationship (spouse, parent, friend, etc.). After completing the network list, they are instructed to successively turn the half pages and rate each listed network member (0–4) on six functional support questions measuring three types of support: affect, affirmation, and aid (see Table 1). Network members' support scores are then summed.

Normative data (*N* = 1,067) [4] shows that the average network size is 10.9 members, but the high standard deviation of this average (5.9) reveals the considerable variability between reported network sizes. This is because participants' network size is dependent on many factors, including how influenced participants are by the presence of 24 spaces as well as the size of their immediate and extended family. For example, family (other than spouse) is the most often listed relationship [2], so a participant with two living parents, a spouse, and four children may list up to seven immediate family members plus supportive siblings, friends, neighbors, and so forth. In contrast, a participant with deceased parents, a spouse, and two children will only have three possible immediate family members to list. 

Because support ratings for each network member are summed, support scores (range = 0–576) vary greatly due to network size alone. Thus, the above participant with seven immediate family members functionally inflates his/her support score. In fact, in the three samples [9–11] used in the present study, network size was very highly correlated with affect scores (.95, .94,  and .95,  resp.) and affirmation scores (.92,  .90,  and .92,  resp.) and a bit less with aid scores (.81,  .82,  and .82,  resp.). As network size increased, support scores increased. 

It is likely that aid's lower correlations with network size are the result of more participants giving some network members aid ratings of 0 than giving 0's for affect or affirmation ratings. When this happens, the participant has effectively dropped that person from their network, and thus reduced the influence of network size on that support score. This happens most often with aid, because some participants list network members who may like (affect) and agree with (affirmation) them but be unable to provide tangible help (aid) such as children, elderly parents, and peripheral network members. In fact, in the present study's third sample [11], where data were entered at this level of specificity (i.e., affect, affirmation, and aid scores from each network member), it was determined that for aid scores, 33% of participants gave a 0 aid rating to at least 1 network member, and 20% of participants gave more than 1 network member a 0 aid rating. In contrast, only 9% of participants gave a 0 affect rating, and 19% gave a 0 affirmation rating. Few participants gave more than one network member a 0 rating for affect or affirmation questions.

In summary, though NSSQ support scores are meant to measure quantity of support, they have two determinants of variability: support ratings and network size. Therefore, raw support scores cannot be taken at face value but should be viewed as support ratings *relative to network size*. For this reason, many investigators remove the influence of network size variability by averaging NSSQ scores, that is, dividing the support score by the network size. 

### 2.2. Averaged Scores

Averaging is not a problem when participants rate all network members uniformly highly or lowly. For example, Participant A lists 7 highly supportive network members, and Participant B lists 14 equally supportive members. B's score (305) is higher than A's (154) only because she listed more network members. However, A's and B's averaged support scores (A (154/7 = 22) and B (305/14 = 21.79)) reflect their support quality relative to their respective network sizes. This is because only their network sizes varied; their support ratings were consistently high. All of their network members uniformly liked them (affect), agreed with them (affirmation), and could help them (aid), but this equality among network members is not typical. 

In a typical NSSQ network, only a few supporters give large amounts of all three types of support and the others contribute in varying degrees, and this reflects reality. That is, some network members make one feel loved and/or are good confidants but cannot offer tangible support and vice versa. This pattern is typified by Participant C: like B, Participant C has a relatively large network (14), but unlike B, her network members' ratings were more varied. C rated 7 network members highly on most support questions but varied the ratings of the other 7 network members giving some high and some low ratings for some types of support. Though both A and C each have 7 highly supportive network members and C has 7 additional network members giving some support, C's averaged score (277/14 = 19.79) is 2.21 points lower than A's (154/7 = 22). Because of variations in her ratings, unlike B, C's numerator (support score) did not keep up with her denominator (network size). 

Of course, it is possible that this “deflation” of averaged scores happens at all network sizes. In fact, if averaging lowered scores consistently for all participants, then lowered scores due to averaging would be the norm and would result in true regressions to the mean. Thus, as is the case with uniformly high or low ratings, if all participants *vary* their support ratings averaging is again not a problem. Norbeck [4] was concerned, however, because participants' support ratings show increased variability with increased network size. That is, the more people one has in his/her network (denominator), the more room for variability of ratings (numerator) and the more chance that one's score will be unduly lowered by averaging if the numerator does not keep pace with the denominator. Indeed, we saw previously that raw support scores increase as network size increases as evidenced by the high positive correlations. Thus, the question is whether averaging results in a statistically significant lowering of (averaged) scores as network size increases. The potential for this is greatest for total network scores and less so for source-specific scores due to the smaller denominators (one relationship category). 

Nevertheless, though Norbeck's [4] concern about the effects of averaging may be warranted, one must also be aware of the effects of using support scores which contain variability due to network size. For example, if high support is related to low stress but support scores are reflective of support as well as network size, it is likely that the size of the relation between support and stress will be reduced. This is due to measurement error or the extraneous variance present in support scores resulting from network size variability. Thus, the risk of making a Type 2 error (failing to detect a real effect) may be greater when using raw support scores, and the deleterious effects of averaging, if any, must be weighed against their beneficial effects in terms of explained variance in the criterion variable.

### 2.3. Research Questions

1. Is there a statistically significant negative correlation between averaged total functional support scores (entire network) and number in network?
2. In addition, are there statistically significant negative correlations between averaged affect, affirmation, and aid scores (entire network) and number in network?
3. Does using averaged total functional support scores provide a measure less infected with extraneous variance and thus produce a more efficient measure than raw total functional support scores? Do averaged total functional support scores yield higher powers than raw scores produced under the same conditions?
4. Similarly, do averaged affect, affirmation, and aid scores provide an analysis with a higher power than respective analyses with raw scores?

## 3. Method

### 3.1. Sample

With institutional review board approval, a secondary analysis was conducted on data from three different samples [9–11] of women who were mothers attending college for their first postsecondary school degree. The same data collection protocol was used in all three studies: participation was invited during a brief in-class presentation of the study, and participants completed the self-administered surveys on their own time and anonymously returned surveys in postage-paid envelopes addressed to the first author. Response rates were high (66%, 45%, and 57%, resp.). Along with the NSSQ, participants completed the Perceived Multiple Role Stress Scale [12] and Role Involvement Questionnaires [13] as well as a demographic data sheet.

All women were community dwelling adults. Table 2 shows that these samples' ((*N* = 157); (*N* = 263); (*N* = 189)) parametric properties are consistent with Norbeck's [4] normative data for community dwelling adult females (*N* = 1,067). Though Norbeck gives no information concerning network size distribution, the majority (75%) of participants in the present study's samples listed ≤14 network members, and 50% listed ≤10-11 members. All samples' total functional support scores were statistically significantly nonnormal, showing positive skews (5.78, 3.32, and 5.60, resp.), and all except sample two showed statistically significantly positive kurtoses (3.5 and 4.54). These findings are consistent with findings concerning normality throughout the NSSQ literature. That is, due to high network size, some participants have very high support scores.

### 3.2. Data Analysis

**Research Questions 1 and 2.**

Using PASW 18 [14] with a.05 alpha level, a bivariate Pearson correlation was computed to determine if there was a statistically significant negative correlation between averaged total functional support scores (summed affect, affirmation, and aid scores from the entire network divided by total number in network) and number in the network. In addition, separate correlations were computed to determine if there were statistically significant negative correlations between averaged affect scores, averaged affirmation scores, and averaged aid scores from the entire network and network number. Because the effect of network size is removed from averaged scores, a significant decrease in averaged scores as network number increases (i.e., a significant negative correlation between averaged scores and network number) would support the claim that averaging scores indeed unduly lowers support scores as network size increases.

**Research Questions 3 and 4.**

In order to answer these research questions, PMRS [12] was used as the criterion variable. PMRS is the amount of role stress experienced by women who are both mothers and students. It has been hypothesized that increased social support is related to decreased levels of PMRS [9–11, 13]. Using the same software and criteria as above, we tested the correlations between PMRS and both raw and averaged total functional support scores and affect, affirmation, and aid scores.

Using G* power 3 [15], we then computed the power of each of the tests given the respective sample sizes, correlations with PMRS of both the various raw and the averaged scores, and *α* = .05. Analogous to the power of a microscope, tests with higher powers can detect finer differences (and thus avoid more Type II errors) than less powerful tests conducted under the same level of statistical rigor. Although there is no established standard for minimally acceptable levels of power, Cohen [16] suggested that power should be at least .80. That is, there is an 80% chance of finding a real, significant effect. Given that averaged scores retain the same information about support quantity as do raw scores but remove the variance associated with differences in network sizes, we expected higher powers among averaged scores than among raw scores.

## 4. Results

**Research Questions 1 and 2**

Results are presented in Table 3. In all three samples, there are no statistically significant decreases in averaged total functional support scores as network size increases. Nor are there statistically significant decreases in averaged affect or affirmation support scores as network size increases. Thus, averaged total functional support scores and averaged affect and affirmation scores do not unduly lower scores as one's network size increases.

However, this is not true for averaged aid scores. In all three samples, there are statistically significant decreases in averaged aid support scores as network size increases. These results are most likely due to the aforementioned high percentage of participants who rated some network members 0 (none at all) for one or both of the aid questions. When participants scored a network member as providing 0 aid, it was most often for network members mentioned later in the network list. That is, it appears that participants begin completing the list of network members by nominating their closest supporters followed by more peripheral supporters. Thus, with the exception of young children and elderly parents, these close supporters are likely able to offer more tangible help than the others. When rating members as providing 0 aid, participants already reduced the influence of network size, and averaging penalized them further, because the denominator was not adjusted to account for this. Averaging does indeed unduly lower aid scores of participants as network number increases.

**Research Questions 3 and 4.**

Results are presented in Table 4. Averaged total functional support scores, averaged affect scores, and averaged affirmation scores resulted in higher powers when correlated with PMRS than their respective raw scores. These results are most dramatic in samples one and three, where powers increased .50 (.47 to .97) and .66 (.13 to .79), respectively. Though sample two's results do not show as marked an improvement, gains in power did considerably improve their probabilities by .24 to .35 points. It should be noted that—all else being equal—larger sample sizes will yield higher power; sample two had 74 more participants than sample one and 106 more participants than sample three. Moreover, we found no statistically significant lowering of averaged scores, as network size increased when investigating research questions 1 and 2 above. Therefore, we recommend using averaged total functional support scores and averaged affect and affirmation scores.

However, averaged aid scores did not perform as well as raw aid scores. The gains in power were modest (.15 and .20) for samples one and three, while power actually decreased in sample two by .19. Thus, in light of results of research questions 1 and 2 showing the statistically significant lowering of averaged aid scores as network size increases and the equivocal effect on power of a test correlation, use of averaged aid scores is not recommended.

## 5. Summary and Conclusions

Averaging reduces the influence of varied network size, but Norbeck [4] was concerned that if support ratings decrease (lower numerators) as network size increases (higher denominators), averaging may unduly lower scores as network size increases. It was found that averaging does not significantly lower total functional support scores or affect and affirmation scores as network size increases. Furthermore, these averaged scores improve analyses by decreasing measurement error as demonstrated by an increase in power. Use of these averaged scores is acceptable, given the underlying nature of the data and the improvements in power.

However, Norbeck's [4] concern about averaging is well founded regarding averaged aid scores. Because network size's influence on aid scores was already reduced by participants' ratings of 0 (none at all) for some network members, averaged aid scores unduly penalize participants as network size increases. Moreover, reduction in measurement error improved only slightly in two samples, and measurement error actually increased in one sample. Use of averaged aid scores is not recommended.

## Figures and Tables

**Table 1:** NSSQ Items.

Functional designation	item
Affect1	How much does this person make you feel liked or loved?
Affect2	How much does this person make you feel respected or admired?
Affirm1	How much can you confide in this person?
Affirm2	How much does this person agree with your actions or thoughts?
Aid1 (short term)	If you needed to borrow $10, a ride to the doctor, or some other immediate help, how much could this person usually help?
Aid2 (long term)	If you were confined to bed for several weeks, how much could this person help you?

**Table 2:** Study samples' parametrics compared with Norbeck's [4] normative data.

NSSQ variables	Norbeck [4] (*N* = 1,067)		Sample 1 (*N* = 157)		Sample 2 (*N* = 263)		Sample 3 (*N* = 189)	
	Mean	*SD*	Mean	*SD*	Mean	*SD*	Mean	*SD*
Network number	10.9	5.9	10.45	5.16	10.93	5.23	11.38	5.19
Affect and affirmation*	127.2	72.7	128.32	69.49	133.16	68.62	137.78	65.71
Aid	53.1	33.4	55.95	28.63	54.42	29.10	58.18	28.82
Total functional support	179.4	102.1	184.56	94.76	187.57	94.21	195.96	91.41

*Note: Norbeck summed affect and affirmation scores in this report.

**Table 3:** Correlations of averaged support scores with network number.

Averaged total functional support	Correlation with network number	*p* value
Sample 1*	−.09	.24
Sample 2**	−.04	.50
Sample 3***	−.08	.26

Averaged affect		
Sample 1	−.08	.34
Sample 2	−.09	.14
Sample 3	−.03	.72

Averaged affirmation		
Sample 1	−.03	.70
Sample 2	−.01	.83
Sample 3	−.01	.93

Averaged aid		
Sample 1	−.24	.003
Sample 2	−.15	.02
Sample 3	−.17	.02

*N = 157,  **N = 263,  ***N = 189.

**Table 4:** Raw and averaged support scores correlations with PMRS and resultant power.

Total functional support	Correlation	*p* value	Power	Change in power
Sample 1*				
Raw scores	−.15	.06	.47	
Averaged scores	−.31	<.0001	.97	↑.50

Sample 2**				
Raw scores	−.15	.02	.68	
Averaged scores	−.21	.001	.93	↑.25

Sample 3***				
Raw scores	−.06	.41	.13	
Averaged scores	−.20	.006	.79	↑.66

*Affect*				
Sample 1				
Raw scores	−.13	.10	.37	
Averaged scores	−.29	<.0001	.96	↑.59

Sample 2				
Raw scores	−.16	.01	.74	
Averaged scores	−.24	<.0001	.98	↑.24

Sample 3				
Raw scores	−.07	.37	.16	
Averaged scores	−.19	.01	.75	↑.59

*Affirmation*				
Sample 1				
Raw scores	−.16	.04	.52	
Averaged scores	−.30	<.0001	.97	↑.45

Sample 2				
Raw scores	−.13	.04	.56	
Averaged scores	−.20	.002	.91	↑.35

Sample 3				
Raw scores	−.07	.38	.16	
Averaged scores	−.21	.003	.83	↑.67

*Aid*				
Sample 1				
Raw scores	−.13	.10	.37	
Averaged scores	−.17	.04	.57	↑.20

Sample 2				
Raw scores	−.13	.03	.56	
Averaged scores	−.10	.12	.37	↓.19

Sample 3				
Raw scores	−.04	.59	.08	
Averaged scores	−.09	.23	.23	↑.15

*N = 157, **N = 263, ***N = 189.
